# Gastrointestinal acute graft versus host disease: a translational perspective from pathogenesis to precision prevention and treatment

**DOI:** 10.3389/fimmu.2026.1876441

**Published:** 2026-07-16

**Authors:** Zhengwei Tan, Jinyu Hu, Baodong Ye, Wenbin Liu

**Affiliations:** 1 Department of Hematology, The First Affiliated Hospital of Zhejiang Chinese Medical University (Zhejiang Provincial Hospital of Chinese Medicine), Hangzhou, China; 2 The First School of Clinical Medicine, Zhejiang Chinese Medical University, Hangzhou, China; 3 Pujiang County Hospital of Traditional Chinese Medicine, Jinhua, China

**Keywords:** acute graft-versus-host disease, fecal microbiota transplantation, gut microbiota, MAGIC biomarkers, ruxolitinib

## Abstract

Allo-HSCT represents a curative option for various hematological disorders. However, aGVHD remains the leading cause of non-relapse mortality following transplantation. The gastrointestinal tract is the most severely affected and prognostically unfavorable target organ in aGVHD, driven by donor T-cell-mediated epithelial damage, microbiota dysbiosis-driven immune amplification, and a self-perpetuating cycle of barrier disruption. Recent multi-omics studies have identified key pathogenic mechanisms, including microbiota-driven MHC-II expression and immunomodulation by microbial metabolites. Biomarker-driven risk stratification using the MAGIC algorithm has shifted management toward precision medicine, while targeted agents such as ruxolitinib, vedolizumab, and microbiota-directed interventions are reshaping therapeutic strategies. Novel interventional modalities, including FMT, recombinant LCN2, and specific bile acids, have forged innovative avenues that synergize microbiota-directed approaches with immunomodulation for the prevention and treatment of GI-aGVHD. This review systematically delineates the latest advances in the pathogenesis, risk stratification, and therapeutic strategies for GI-aGVHD, and envisions future directions for precision medicine centered on personalized microbiota-immune interventions.

## 1 Introduction

Allogeneic hematopoietic stem cell transplantation (allo-HSCT) represents a potentially curative therapeutic strategy for a range of hematologic disorders, including acute leukemia and aplastic anemia (AA). However, acute graft-versus-host disease (aGVHD) remains a central clinical challenge following transplantation (1). Grade II-IV aGVHD occurs in 35%-46% of patients by 6 months, with nearly half being steroid-refractory, contributing significantly to transplant-related mortality (2, 3). Although the skin is the most commonly affected organ in aGVHD, the gastrointestinal (GI) tract represents the most severely affected target organ, exhibiting the poorest prognosis and the highest rate of non-relapse mortality among all involved sites (4).

The classical three-phase model of aGVHD pathogenesis has been substantially expanded by recent investigations revealing the central orchestrating role of the intestinal microenvironment (5–7). Reduced microbial diversity, depletion of specific commensal taxa (such as *Blautia* spp.), and the concurrent expansion of opportunistic pathogens (such as *Enterococcus faecalis*) has been convincingly associated with both the onset and severity of aGVHD (6, 8, 9). Notably, emerging evidence reveals a vicious cycle between dysbiosis and epithelial injury. Disruption of the intestinal barrier facilitates the translocation of bacterial products, which fuels systemic and local immune activation. In turn, activated T cells secrete inflammatory cytokines such as IFN-γ that directly compromise epithelial integrity and alter the luminal redox milieu, thereby further suppressing the growth of obligate anaerobes (10, 11). Consequently, strategies that restore eubiosis and reinforce epithelial barrier function are now essential to interrupt this cycle and mitigate T-cell-mediated injury.

In recent years, the application of high-throughput multi-omics and spatial transcriptomics technologies has enabled the delineation of the cellular and molecular landscape of GI-aGVHD. These advances have led to the identification of novel regulatory cell subsets, including Lipocalin-2 (LCN2)^+^ regulatory neutrophils and type 2 innate lymphoid cells (ILC2s), and have elucidated the molecular mechanisms by which microbial metabolites such as short chain fatty acids and secondary bile acids modulate host immunity (12–15). In clinical translation, an integrated risk score based on the Mount Sinai Acute GVHD International Consortium (MAGIC) biomarker framework has markedly improved the prediction of non-relapse mortality (NRM) and facilitated the recognition of low-risk patient cohorts who may benefit from reduced-intensity immunosuppressive strategies (16, 17). Concurrently, the therapeutic strategy is transitioning from an era dominated solely by corticosteroids to one characterized by diversified and precision-targeted interventions. Emerging therapies such as ruxolitinib (18), vedolizumab (19), and fecal microbiota transplantation (FMT) (20, 21) are reshaping the aGVHD treatment landscape. This review summarizes recent advances in GI-aGVHD pathogenesis, risk stratification, and therapeutics, focusing on the microbiota-immune axis and biomarker-driven precision medicine.

## 2 New perspectives on the pathogenesis of GI-aGVHD

### 2.1 Revisiting the classical three-phase model

**Figure 1:**
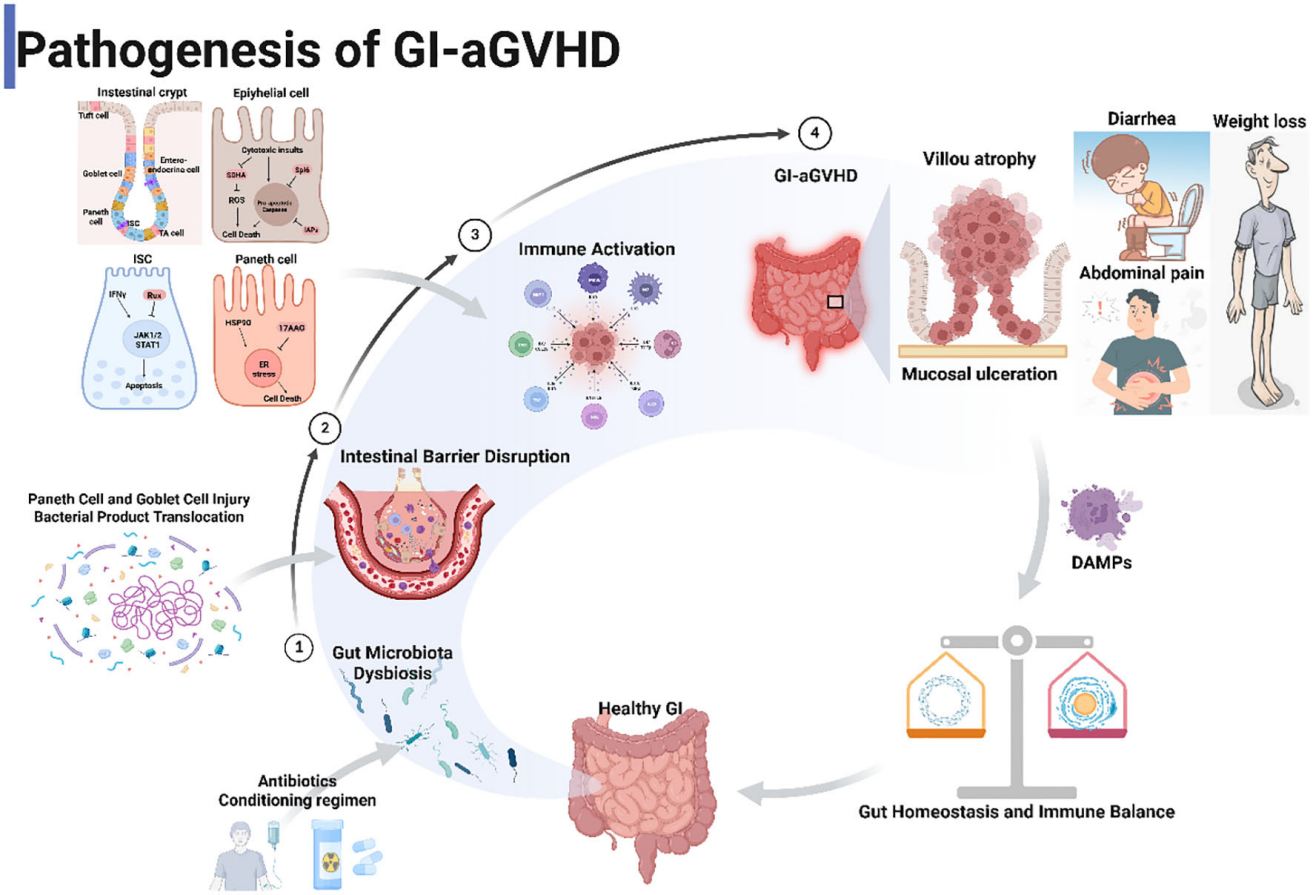
Pathogenesis of GI-aGVHD. This schematic illustrates the four-stage cascade driving GI-aGVHD after allo-HSCT. Conditioning regimens and antibiotics disrupt the symbiotic microbiota and compromise mucosal barrier integrity, enabling bacterial product translocation into the lamina propria. There, luminal antigens activate host dendritic cells and macrophages, which prime donor-derived alloreactive T cells and drive Th1/Th17 polarization. The resulting pro-inflammatory milieu (IFN-γ, TNF-α, IL-6, IL-17) precipitates JAK2/STAT3- and MS4A3-mediated crypt apoptosis, intestinal stem cell exhaustion, and Paneth/goblet cell depletion. The cascade comprises (1): Gut Microbiota Dysbiosis (2); Intestinal Barrier Disruption (3); Immune Activation; and (4) GI-aGVHD Manifestation, presenting as villous atrophy, mucosal ulceration, and severe diarrhea, abdominal pain, and weight loss. Tissue destruction in stage 4 releases DAMPs that perpetuate microbial dysbiosis through a self-amplifying feed-forward loop, driving disease chronicity and steroid resistance.

The classical paradigm of aGVHD pathogenesis comprises three sequential phases (7, 22, 23). Phase I involves conditioning-induced tissue injury and inflammatory cytokine storm, which collectively leads to activation of host antigen-presenting cells (APCs). Phase II encompasses donor T-cell activation and proliferation upon recognition of host allo-antigens. Finally, Phase III is characterized by the attack of target organ tissues by effector T cells and the inflammatory cytokines they secrete. While this framework remains central to our understanding of aGVHD, recent investigations have unveiled novel nuances within each phase, delineating a more intricate regulatory network and revealing potential interventional targets (**Figure 1**).

First, conditioning-induced tissue damage is not merely a passive event. Myeloablative conditioning regimens cause intestinal epithelial injury that triggers the release of abundant damage-associated molecular patterns (DAMPs) and pathogen-associated molecular patterns (PAMPs) (1, 24, 25). Concurrently, direct cytotoxicity from the conditioning regimen and dysbiosis driven by broad-spectrum antibiotics converge to disproportionately target Paneth and Goblet cells. Owing to their high secretory burden and rapid turnover, these cells are exquisitely vulnerable to metabolic and oxidative stress, which culminates in depleted antimicrobial peptide secretion and disintegration of the protective mucus barrier (26, 27). These DAMPs and PAMPs not only directly activate host APCs but also promote the production of pro-inflammatory cytokines such as IL-1β through NLRP3 inflammasome activation, thereby establishing an inflammatory milieu conducive to subsequent donor T-cell activation.

Second, donor T-cell activation does not rely exclusively on host APCs. Emerging evidence indicates that donor-derived cDC subsets, particularly XCR1^+^ and CD11b^+^ cDCs, are key amplifiers of GI-aGVHD (28, 29), driving Th1 and Th17 polarization, respectively. This activation cascade is further fueled by the translocation of luminal bacterial products, such as lipopolysaccharide (LPS), across the disrupted epithelial barrier, which triggers a potent Th1/Tc1 cytokine storm dominated by IFN-γ and TNF-α. The ensuing inflammation drives apoptosis of intestinal crypt base cells and widespread mucosal denudation (30, 31). This insight challenges the traditional dichotomy of “host APC priming followed by donor APC amplification” and suggests that targeting specific donor cDC subsets, rather than globally depleting APCs, may constitute a more precise therapeutic strategy.

Finally, effector T-cell assault on target organs is not unidirectional. In the intestine, IFN-γ and TNF-α secreted by T cells not only directly induce enterocyte apoptosis but also elevate luminal oxygen tension by suppressing mitochondrial oxidative phosphorylation in intestinal epithelial cells, thereby disrupting the niche of obligate anaerobes and exacerbating gut dysbiosis (32, 33). The resulting clinical syndrome, characterized by severe diarrhea, abdominal pain, and progressive weight loss, further compromises barrier integrity and perpetuates the state of dysbiosis, establishing a pathogenic positive feedback circuit.

### 2.2 Gut microbiota: from bystander to active driver

The role of the gut microbiota in aGVHD has progressed from correlative studies to the validation of causal mechanisms. Factors including conditioning regimens, broad-spectrum antibiotics and dietary alterations can induce gut dysbiosis, characterized by a loss of microbial diversity, depletion of beneficial anaerobic commensals (such as *Blautia* spp., *Faecalibacterium* spp.), and the overgrowth of potentially pathogenic bacteria (such as *Enterococcus* spp., *Akkermansia muciniphila*) *(*34–36). Clinical studies have established that higher microbial diversity, particularly during the neutrophil engraftment period, is associated with lower post-transplant mortality, serving as a robust independent protective predictor (37, 38).

#### 2.2.1 The *Enterococcus*-intestinal epithelial MHC-II axis in GI-GVHD pathogenesis

Utilizing MHC-mismatched murine transplant models and gnotobiotic single-colonization techniques, Nguyen (9) demonstrated that the overgrowth of endogenous *Enterococcus* is closely associated with elevated colonic epithelial MHC-II expression and increased mortality. *E. faecalis* colonization induces IFN-γ production by CD4^+^ T cells and NK cells, which upregulates epithelial MHC-II expression, amplifying local antigen presentation and T-cell-mediated inflammation. Therapeutically, introduction of a nisin-producing *Blautia producta* strain can prevent enterococcal predominance and improve survival in GVHD, providing a theoretical basis for precise microbiome-based intervention.

#### 2.2.2 Protective mechanisms of commensal *Bacteroides*

In contrast to pathogenic bacteria, specific commensal consortia confer protection. Research by a Chinese team revealed that the abundance of *Bacteroides* is inversely correlated with the incidence of GI-aGVHD, with its protective effects dependent on the Type VI Secretion System (T6SS) (39). T6SS-mediated antagonism modulates the composition and metabolome of the gut microbiota, particularly affecting bile acid metabolism. By inhibiting the accumulation of primary bile acids (such as chenodeoxycholic acid), this mechanism reduces T cell activation and preserves intestinal barrier integrity. This discovery not only elucidates a microbiota-bile acid-T cell regulatory axis but also provides a proof-of-concept for engineering T6SS-enhanced bacterial strains with augmented therapeutic capacity. Conversely, Hayase demonstrated that mucus-degrading *Bacteroides* species can exacerbate GVHD when expanded by carbapenem exposure (40), whereas *Bacteroides* ovatus specifically alleviates dysbiosis-induced GVHD through competitive exclusion mechanisms (41). These findings underscore the species-specific and context-dependent nature of commensal bacterial effects in GVHD pathogenesis.

#### 2.2.3 Roles of the virome and mycobiome

Beyond bacteria, the contributions of the gut virome and mycobiome to aGVHD pathogenesis are increasingly recognized. Thiele Orberg (42) reported that bacteriophages associated with *Lachnospiraceae* and *Oscillospiraceae* carry genes encoding butyrate synthesis enzymes, and their abundance correlates positively with levels of immunomodulatory metabolites and patient survival. Another study found that post-transplant expansion of *Candida* species, particularly the *Candida parapsilosis* complex, is associated with an increased risk of aGVHD and transplant-related mortality (43). These findings underscore that multi-kingdom microbial interactions (bacteria, viruses, and fungi) collectively shape the ecological foundation underlying aGVHD.

### 2.3 Disruption of the intestinal barrier and increased permeability

The integrity of the intestinal epithelial barrier constitutes the first line of defense against aGVHD. Dysbiosis compromises the intestinal mucosal barrier through multiple mechanisms. Depletion of short-chain fatty acid (SCFA)-producing bacterial taxa lead to downregulated expression and aberrant localization of tight junction proteins, thereby increasing intestinal epithelial permeability. In parallel, excessive proliferation of pathogenic bacteria directly induces apoptosis of intestinal epithelial cells and destruction of crypt architecture (44–46). Studies have demonstrated that MLCK210-mediated loss of intestinal epithelial barrier function is a critical driver of GVHD progression (47), and that inhibition of MLCK210-dependent barrier regulation may represent an effective strategy to limit disease advancement.

At a more refined pathological level, the spatial atlas of human GI-aGVHD constructed by Azulay (13) has revealed increased fibrosis, abnormal crypt morphology, loss of Paneth cells, and aberrant accumulation of endocrine cells within the crypts in aGVHD patients. Notably, host-derived cells remain dominant within the intestinal plasma cell and T-cell compartments for an extended period post-transplantation, suggesting that in addition to donor-derived T cells, other cell types, such as host-derived macrophages and neutrophils, play significant roles in the inter-individual progression of aGVHD.

Furthermore, T-cell-mediated mitochondrial dysfunction in intestinal epithelial cells drives a shift in luminal oxygen tension from an anaerobic toward a microaerophilic state. This directly suppresses the growth of obligate anaerobes, including butyrate-producing bacteria, while facilitating the expansion of facultative anaerobes (such as *Enterococcus* spp.), thereby establishing a self-perpetuating vicious cycle in which immune injury leads to loss of hypoxia, which in turn triggers dysbiosis and ultimately exacerbates inflammation (48).

### 2.4 Key mediating roles of metabolic products

Microbiota-derived metabolites serve as central molecular bridges linking dysbiosis to host immune responses.

#### 2.4.1 Short-chain fatty acids

SCFAs (butyrate, propionate, acetate), produced by bacterial fermentation of dietary fiber, enhance the expression of epithelial tight junction proteins and promote Tregs differentiation, exerting anti-inflammatory and barrier-protective effects. Dysbiosis-induced reduction in SCFA production results in diminished Treg function and excessive activation of pro-inflammatory cells such as CD8^+^ T cells (49, 50). Butyrate restores intestinal histone acetylation, suppresses apoptotic gene expression, and mitigates aGVHD severity in preclinical models (51).

Clinical translation of SCFA-based interventions remains in its infancy. To date, only one prospective study has evaluated oral sodium butyrate administration from engraftment to day 90-100, demonstrating merely a non-significant trend toward reduced GI-aGVHD incidence without meeting the primary endpoint (52). The scarcity of clinical data reflects several translational challenges, including poor oral bioavailability of SCFAs, rapid colonic metabolism, and lack of standardized dosing regimens. Pharmacokinetic constraints limit therapeutic efficacy, as poor oral bioavailability and rapid proximal absorption restrict colonic delivery where SCFAs exert their principal effects. And the efficacy of exogenous butyrate relies on a receptive microbial ecosystem, yet conditioning regimens profoundly deplete butyrate-producing taxa such as *Faecalibacterium* and *Roseburia*, which potentially renders supplementation ineffective or even counterproductive in the early post-transplant period. Finally, the protective window for SCFAs may be narrow, with stage-dependent effects rather than uniform benefit across transplant phases (53). Notably, Golob (54) reported that patients retaining higher levels of butyrate-producing bacteria after GVHD onset paradoxically exhibited an increased risk of steroid-refractory disease. A recent study by Riwes (55) offers a complementary strategy: dietary resistant potato starch (RPS) significantly elevated fecal butyrate levels and stabilized plasma metabolites at engraftment in allo-HCT recipients. These findings underscore that SCFA-based interventions, if pursued clinically, will likely require precision delivery systems (such as pH-dependent colonic release formulations, butyrate-producing live biotherapeutic products) and biomarker-guided temporal stratification to identify optimal intervention windows, rather than uniform supplementation across all transplant phases.

#### 2.4.2 Bile acids

Primary bile acids are converted into secondary bile acids (such as ursodeoxycholic acid, tauroursodeoxycholic acid, lithocholic acid) by the gut microbiota. Secondary bile acids inhibit the secretion of pro-inflammatory cytokines by activating the TGR5 receptor or suppressing the FXR signaling pathway (56). Lindner (57) found that the gene abundance of bile salt hydrolase and 7α-dehydroxylase in the gut microbiota of aGVHD patients is markedly reduced, leading to secondary bile acid deficiency, whereas UDCA supplementation restores bile acid homeostasis and attenuates T cell-driven inflammation. Haring (58) confirmed that TUDCA alleviates aGVHD by downregulating MHC-II expression on intestinal epithelial cells while preserving the graft-versus-leukemia effect.

#### 2.4.3 Tryptophan metabolites

Indole derivatives (such as indole-3-propionic acid, indole-3-carboxaldehyde) promote IL-22 secretion through activation of the aryl hydrocarbon receptor (AhR), thereby enhancing intestinal mucosal repair capacity (59). Swimm (60) demonstrated that specific indole derivatives limit radiation-induced damage and promote epithelial regeneration via type I interferon signaling. However, the role of IL-22 is dualistic. In steroid-refractory aGVHD (SR-aGVHD), the accumulation of donor-derived IL-22^+^ T cells, along with IL-22-dependent dysbiosis and loss of anti-inflammatory CX3CR1 mononuclear phagocytes, collectively drives the refractory state of the disease (61).

### 2.5 Expansion of the immune regulatory cell network

#### 2.5.1 Dysfunction of Treg cells

Tregs play a central role in maintaining intestinal immune tolerance. In aGVHD, circulating Tregs are reduced in number and suppressive function, with an expansion of inflammatory CD45RA^-^FoxP3^ep^ subsets that produce TNF-α (62). This phenomenon is further corroborated by the quantitative deficiency of mucosal FOXP3^+^ Tregs observed in aGVHD lesions (63). As detailed in the **Table 1**, the diminished Tregs population coupled with the emergence of functionally aberrant inflammatory subsets directly contributes to the breakdown of immune tolerance and the pathogenesis of aGVHD.

**Table 1:** Summary of key pathogenic mechanisms of GI-aGVHD.

Mechanistic level	Specific mechanism/key molecules/cells	Pathogenic or protective effect	Clinical or preclinical evidence
Microbiota dysbiosis	Microbiota diversity ↓, expansion of *Enterococcus*, *Akkermansia muciniphila*	Pathogenic: Promotes inflammation, degrades mucus layer, disrupts barrier	Peled et al., NEJM 2020 (37)
	*Depletion of butyrate-producing bacteria such as Blautia, Faecalibacterium*	Pathogenic: Decreased SCFAs, impaired Treg function	Jenq et al., BBMT 2015 (8); Meedt et al., CID 2022 (53)
	*Colonization of Bacteroides ovatus (xylose production)*	Protective: Inhibits mucus-degrading bacteria, alleviates GVHD	Hayase et al., Cell 2022 (40); Hayase et al., Cell Host Microbe 2024 (41)
	Enterococci induce intestinal epithelial MHC-II expression via IFN-γ	Pathogenic: Amplifies local antigen presentation and T cell activation	Nguyen et al., Blood 2026 (9)
Intestinal barrier disruption	Loss of tight junction proteins (MLCK210 pathway)	Pathogenic: ↑ Permeability, translocation of bacterial products	Nalle et al., JCI 2019 (47)
	Loss of Paneth cells → Decreased α-defensin	Pathogenic: Exacerbates dysbiosis, ↑ bacterial translocation	Eriguchi et al., Blood 2012 (27)
	Loss of goblet cells → Thinning of mucus layer	Pathogenic: Direct bacterial-epithelial contact, amplifies inflammation	Ara et al., Sci Transl Med 2020 (69)
	Loss of luminal hypoxia → ↑ Oxygen concentration	Pathogenic: Suppresses obligate anaerobes, promotes expansion of facultative anaerobes	Seike et al., Immunity 2023 (48)
Metabolic abnormalities	↓ SCFAs (Butyrate, Propionate)	Pathogenic: IEC energy deficiency, reduced HDAC inhibition, ↓ Treg differentiation	Mathewson et al., Nat Immunol 2016 (51)
	↓ Secondary Bile Acids (UDCA, TUDCA, LCA)	Pathogenic: Attenuated TGR5/FXR signaling, ↑ pro-inflammatory cytokines	Lindner et al., Nat Microbiol 2024 (57)
	↓ Tryptophan Metabolites (Indoles)	Pathogenic: Insufficient AhR activation, ↓ IL-22 production, impaired epithelial repair	Swimm et al., Blood 2018 (60)
	↑ TMAO	Pathogenic: Induces M1 macrophage polarization, exacerbates inflammation	Wu et al., Blood 2020 (70)
Immune cell dysregulation	↓ Treg numbers + Functional abnormalities (Inflammatory Treg subsets)	Pathogenic: Breakdown of immune tolerance	Jarosch et al., Cell Rep Med 2023 (71); Seong et al., Korean J Intern Med 2025 (62)
	Expansion of LCN2^+^ regulatory neutrophils	Protective: Promotes IL-10 production by macrophages via LCN2	Czech et al., Sci Transl Med 2024 (64)
	Adoptive transfer of donor ILC2s	Protective: Secretes IL-22, promotes epithelial repair	Bruce et al., J Clin Invest 2017 (65)
	↑ Intestinal epithelial MHC-II expression (Microbiota-driven)	Pathogenic: Directly activates CD4^+^ T cells, initiates GVHD	Koyama et al., Immunity 2019 (72)

#### 2.5.2 Discovery of novel regulatory immune cell subsets

Recent studies have unveiled previously underappreciated subsets of regulatory immune cells. Czech (64) utilized single-cell RNA sequencing to identify a neutrophil subset expressing LCN2 that expands in a murine model of GI-aGVHD. This subset promotes a tolerogenic effect by enhancing macrophage IL-10 production and reducing MHC-II expression via the LCN2-SLC22A17-IGF-1R axis. Notably, treatment with recombinant LCN2 alleviated aGVHD severity without compromising the graft-versus-leukemia (GVL) effect. Another study identified donor-derived type 2 innate lymphoid cells (ILC2s) as capable of preventing and treating lower GI-aGVHD while preserving GVL activity (65), thereby offering a novel direction for developing adoptive cell therapies based on innate immune cells. Furthermore, intestinal epithelium-derived IL-34 has been identified as a tissue-intrinsic cytokine that mitigates GI-GVHD severity by reprogramming donor macrophages toward an anti-inflammatory phenotype, a protective effect dependent on the expression of Apolipoprotein E (APOE) in donor macrophages (66).

#### 2.5.3 Role of T cell-intrinsic regulatory factors

Research led by Huang (67, 68) at Peking University has elucidated the critical role of SOCS1 as a key checkpoint governing T cell pathogenicity. T cell-specific deletion of SOCS1 reshapes the immune microenvironment via the STAT1/2-CCL5 axis, driving the differentiation of CD8^+^ T cells toward a pathogenic phenotype and exacerbating aGVHD. These findings suggest that SOCS1 may serve as a predictive biomarker and therapeutic target for aGVHD. However, given the potential context-dependent roles of SOCS1 across different immune cell subsets, its clinical translation warrants careful and rigorous evaluation.

## 3 Diagnosis and risk stratification: from clinical grading to biomarker-driven precision medicine

### 3.1 Limitations of traditional grading systems

The diagnosis of GI-aGVHD continues to rely primarily on clinical manifestations, including stool volume, nausea/vomiting, abdominal pain, which supplemented by histopathological confirmation via endoscopic biopsy in routine practice at most transplant centers. However, grading systems based solely on clinical symptoms, such as the Minnesota risk classification, have notable limitations. They distinguish only between standard-risk and high-risk categories, lacking a low-risk stratum that could guide immunosuppression de-escalation strategies. Furthermore, the subjectivity inherent in symptom assessment leads to substantial heterogeneity in both prognostication and evaluation of treatment response.

### 3.2 Development and validation of the MAGIC biomarker algorithm

The Mount Sinai Acute GVHD International Consortium (MAGIC) developed a biomarker-driven risk algorithm based on soluble ST2 and REG3α, both reflecting gastrointestinal tissue damage and immune-mediated crypt destruction, termed the MAGIC Algorithm Probability (MAP) (73–75). Akahoshi (16) subsequently integrated MAP with clinical Minnesota risk into the MAGIC Composite Score (MCS), which improved 6-month NRM prediction and identified a low-risk cohort (~40%) potentially eligible for glucocorticoid-sparing strategies. Building upon this framework, subsequent research has proposed the MAGIC composite response (MCR) as a novel clinical trial endpoint that synthesizes clinical and biomarker data at day 28, thereby further refining outcome prediction and correctly reclassifying 30% of patients whose initial assessment relied solely on clinical response (17). This reclassification underscores the need for biomarker-integrated endpoints in future trial designs. The specific application scenarios and clinical value of the aforementioned biomarkers in risk prediction, diagnostic grading, prognostic assessment, treatment response monitoring, and guidance for tapering immunosuppression in GI-aGVHD are systematically summarized in **Table 2**.

**Table 2:** Application of biomarkers in risk stratification and treatment guidance for GI-aGVHD.

Application scenario	Biomarker name	Sample type	Clinical significance and decision-making value
Risk prediction (pre-transplant/early phase)	Microbiota Diversity (16S Sequencing) (37)	Stool	Low diversity during neutrophil engraftment → 2-fold increase in NRM risk; guides enhanced monitoring or prophylactic intervention.
	*Enterococcus Domination (*9)	Stool	Independent predictor of GVHD-related mortality; guides antibiotic selection (avoidance of broad-spectrum anti-anaerobic antibiotics).
Diagnosis & grading	sST2 + REG3α (MAP) (73–77)	Serum	Quantifies GI tissue damage; superior to clinical grading alone; guides necessity of endoscopic biopsy.
Prognosis assessment	MAGIC Composite Score (MCS) (16)	Serum + Clinical	Integrates Ann Arbor risk with biomarkers; predicts 6-month NRM with AUC of 0.76; identifies low-risk group (~40%).
Treatment response monitoring	MAGIC Composite Response (MCR) (17)	Serum + Clinical	Assessed at Day 28; correctly reclassifies 30% of clinical “non-responders” or “responders”; may serve as a surrogate for traditional endpoints.
Intestine-specific prognosis	REG3α、AREG (78, 79)	Serum	Predicts short- and long-term GI GVHD outcomes; superior to systemic inflammatory markers (such as CRP).
Guidance for tapering immunosuppression	MCS Low-Risk Group (~40% of patients) (16, 76)	Serum + Clinical	May help avoid prolonged corticosteroid therapy and related complications; guides individualized tapering strategies.

### 3.3 Distinct value of gut-specific biomarkers

Gut-specific biomarkers demonstrate superiority over systemic biomarkers in predicting both short- and long-term aGVHD outcomes. A study of 715 patients with newly diagnosed aGVHD revealed that any combination of REG3α, ST2, and AREG successfully stratified patients according to NRM risk (80). Additionally, molecular imaging modalities such as ¹^8^F-FLT PET are being explored for the non-invasive identification of GI-aGVHD and for guiding targeted biopsy in anatomically challenging locations (81, 82). The establishment of these multimodal biomarker platforms is driving a paradigm shift in the management of GI-aGVHD from empirical therapy toward risk-stratified, precision-guided intervention.

## 4 Evolution of treatment strategies: from the corticosteroid era to precision targeting

The management of GI-aGVHD has undergone a fundamental transformation, evolving from an era dominated by empirical corticosteroid use toward a diversified therapeutic landscape encompassing targeted immunomodulation, microbiota-directed interventions, and biomarker-guided precision approaches. This section systematically delineates the current therapeutic framework, distinguishing between established standards of care and emerging strategies at various stages of clinical development, while explicitly differentiating prophylactic from treatment-oriented interventions. The categories, mechanisms of action, and levels of evidence for emerging therapeutic strategies for intestinal acute graft-versus-host disease are detailed in **Table 3**.

**Table 3:** Emerging therapeutic strategies for GI-aGVHD and their evidence base.

Category	Intervention	Mechanism of action	Key clinical/preclinical evidence	Level of evidence
Novel prophylactic strategies	Vedolizumab (19)	Anti-α4β7 integrin antibody; blocks homing of gut-tropic T cells	Lower GI-aGVHD event-free survival 85.5% vs 70.9% (HR 0.45, *P* < 0.001)	Level 1 (Phase III RCT)
	Ruxolitinib (18, 94)	JAK1/2 inhibitor; suppresses pro-inflammatory cytokine signaling	Grade II-IV aGVHD 0% vs 15.8%; 1-year GRFS 91.6% vs 72.1%	Level 1 (Phase III RCT)
	UDCA (95)	Inhibits FXR; restores bile acid homeostasis; anti-inflammatory	Reduced incidence of hepatic and intestinal GVHD	Level 2 (RCT)
	Resistant starch/FOS (55)	Promotes growth of butyrate-producing bacteria; increases SCFAs	Reduced aGVHD incidence; well tolerated	Level 2 (Phase II)
	SER-155 (97)	Defined bacterial consortium; competitive inhibition of enteric pathogens	Safe; reduced dominance of pathogenic bacteria	Level 1 (Phase I)
Emerging therapies for SR-GVHD (clinical stage)	MaaT013 (98)	Pooled, high-richness microbiota biotherapeutic; restores microbial diversity and SCFA production	GI-ORR 62% (CR 38%, VGPR 20%); 1-year OS 54% in ruxolitinib-refractory patients	Level 1 (Phase III Single-arm)
	F-652 (rhIL-22) (99, 100)	Recombinant human IL-22 fusion protein; promotes epithelial regeneration and antimicrobial peptide secretion	Day 28 response 70% in newly diagnosed lower GI-aGVHD; case reports of efficacy in ruxolitinib-refractory disease	Level 2 (Phase II)
	Xenopax (anti-CD25) (101)	Humanized IL-2 receptor antagonist; blocks IL-2 signaling and T-cell activation	RELAX multicenter retrospective study: Effective and well tolerated in real-world SR-aGVHD; approved as Class II biologic	Level 2 (Multicenter Retrospective)
JAK1 selective inhibition	Itacitinib (91, 92)	Selective JAK1 inhibitor; reduces pro-inflammatory cytokines with less myelosuppression than JAK1/2 inhibitors	ORR 89% in low-risk aGVHD; steroid-sparing in 79% of patients; fewer serious infections	Level 2 (Phase II)
Anti-CD6 immunomodulation	Itolizumab (103, 104)	Anti-CD6 mAb; blocks CD6-ALCAM pathway, downregulates Teffs while preserving Tregs	Day-99 CR 44.9% vs. 28.6%; median DOR 336 vs. 72 days; program discontinued	Level 1 (Phase III)
Serine protease inhibition	α1-Antitrypsin (105–107)	Anti-inflammatory serpin; suppresses IL-32, TNF-α, IL-1β; anti-apoptotic	ORR 65% in SR-aGVHD; well tolerated	Level 2 (Phase II)
Mesenchymal stem cells	Remestemcel-L (111, 112)	Immunomodulatory and tissue-repair properties; promotes Treg expansion; regulates multiple immune lineages	ORR 70.4%; FDA-approved (2024) for pediatric SR-aGVHD; adult meta-analysis: ORR RR 1.13	Level 1 (Phase III)
Preclinical therapeutic targets	Recombinant LCN2 (64)	LCN2-SLC22A17-IGF-1R axis; promotes IL-10 production by macrophages	Murine model: Alleviates aGVHD without impairing GVL effect	Preclinical
	R-spondin1 (108, 109)	Wnt agonist; promotes regeneration of intestinal stem cells and Paneth cells	Murine model: Attenuates GVHD; restores microbial diversity	Preclinical
	Teduglutide (GLP-2 analog) (110, 115)	Promotes expansion of intestinal stem cells and Paneth cells	Improves microbial diversity; alleviates intestinal injury	Preclinical
	TUDCA (Tauroursodeoxycholic Acid) (58)	Activates TGR5; inhibits IEC MHC-II expression; anti-apoptotic	Alleviates aGVHD while preserving GVL effect	Preclinical
	Butyrate/Propionate (51)	HDAC inhibition; promotes Treg differentiation; energy source for IECs	Oral or enema administration alleviates GVHD	Preclinical

### 4.1 First-line therapy: central role and limitations of glucocorticoids

Glucocorticoids (such as methylprednisolone) remain the standard first-line therapy for aGVHD, as endorsed by the updated EBMT consensus recommendations (83, 84). Despite recent advances in immunoprophylaxis strategies, the incidence of aGVHD still ranges from 20% to 80%, and only approximately 60% of affected patients achieve a response to first-line corticosteroid therapy (85). The prognosis for patients with SR-aGVHD is exceedingly poor, with markedly elevated mortality rates, particularly among those with gastrointestinal involvement (86). Consequently, the development of effective second-line and subsequent therapeutic regimens has remained a central focus of clinical investigation.

### 4.2 Paradigm shift in second-line therapy

The advent of ruxolitinib, a JAK1/2 inhibitor, has fundamentally reshaped the therapeutic landscape for SR-aGVHD, consistent with EBMT recommendations that endorse the addition of novel targeted agents for steroid-refractory disease (84). Ruxolitinib acts by inhibiting the JAK/STAT signaling pathway, thereby reducing the release of proinflammatory cytokines (such as IL-6, IFN-γ) and modulating T-cell function while preserving the GVL effect (18, 87). Clinical trial evidence demonstrates that ruxolitinib yields superior aGVHD improvement rates compared with standard therapy and is associated with a reduced risk of treatment failure (18, 88–90). Based on these data, ruxolitinib has been established as the standard second-line therapy for SR-aGVHD and is now incorporated into international treatment guidelines.

Beyond ruxolitinib, itacitinib, a selective JAK1 inhibitor, represents another promising JAK-targeted strategy with a potentially more favorable safety profile. In a multicenter phase II trial, 70 low-risk aGVHD patients received itacitinib monotherapy (200 mg/d) for 28 days and were compared with 140 matched controls receiving systemic corticosteroids. Itacitinib achieved comparable day-28 overall response (89% vs. 86%, *P* = 0.67), with significantly faster responses by day 7 (81% vs. 66%, *P* = 0.02) and fewer serious infections within 90 days (27% vs. 42%, *P* = 0.04). Notably, 79% of patients never required corticosteroids for aGVHD management (91). These findings support itacitinib as a potential steroid-sparing alternative for low-risk aGVHD; however, the subsequent phase III trial failed to demonstrate benefit when itacitinib was added to corticosteroids in unselected populations (92).

### 4.3 Novel prophylactic strategies for GI-aGVHD

Beyond the established calcineurin inhibitor-based prophylaxis regimens, several novel strategies have demonstrated efficacy in preventing GI-aGVHD, offering the potential to reduce the incidence of severe disease before it occurs.

#### 4.3.1 Gut-selective T-cell trafficking blockade

Vedolizumab is a gut-selective monoclonal antibody targeting α4β7 integrin that attenuates intestinal inflammation by inhibiting the migration of gut-homing T lymphocytes (93). In a randomized, double-blind, placebo-controlled phase III trial reported by Chen (19), the addition of vedolizumab to standard calcineurin inhibitor-based GVHD prophylaxis significantly improved lower GI-aGVHD survival compared with placebo. The incidence of treatment-related serious adverse events was similar between the two groups. These findings provide robust evidence supporting targeted prophylaxis for GI-aGVHD, and the gut-selective nature of vedolizumab theoretically circumvents the broad adverse effects associated with systemic immunosuppression.

#### 4.3.2 JAK inhibition in prophylaxis

A prospective phase II trial conducted by Zhang (94) provided the evidence that the addition of ruxolitinib to standard GVHD prophylaxis significantly reduces the risk of grade II-IV aGVHD in patients with AA undergoing allo-HSCT. Notably, no grade III-IV severe aGVHD occurred in the experimental group, compared with an incidence of 15.8% in the control group. Furthermore, the ruxolitinib group exhibited accelerated platelet engraftment, enhanced Tregs recovery, and a significantly improved 1-year GRFS (91.6% vs. 72.1%). These findings offer valuable insights for optimizing prophylactic strategies in high-risk aGVHD populations.

#### 4.3.3 Microbiota-directed prophylaxis

Several microbiota-based strategies have demonstrated therapeutic potential in the prophylactic setting. Ursodeoxycholic acid (UDCA), a secondary bile acid with anti-inflammatory properties, was assessed in a randomized controlled trial, which demonstrated a reduced incidence of both hepatic and intestinal GVHD (95). Dietary interventions, notably those incorporating resistant starch and fructo-oligosaccharides (FOS), have proven feasible and shown preliminary efficacy in modulating microbial metabolism and attenuating aGVHD incidence, as evidenced by Phase II studies (96). Furthermore, live biotherapeutic products, such as SER-155, have established a favorable safety profile in Phase I trials and are presently undergoing further clinical evaluation (97).

### 4.4 Emerging therapies for steroid-refractory GI-aGVHD

For patients who fail both corticosteroids and ruxolitinib, a range of emerging therapies at various stages of clinical development offer potential therapeutic avenues. These approaches can be categorized by their mechanism of action and level of clinical evidence.

#### 4.4.1 Microbiota restoration therapies

Fecal microbiota transplantation (FMT) represents a broad-spectrum intervention aimed at restoring gut microbial diversity. While a randomized trial in AML/allo-HSCT patients showed that third-party FMT improved microbial diversity, unexpectedly associated with a higher incidence of aGVHD, underscoring the complexity of post-transplant microbial manipulation (20). Within the specific milieu of post-transplant alloimmune activation, commensal bacteria that are ordinarily beneficial to the host may inadvertently stimulate alloreactive immune cells through the presentation of donor-derived antigens, thereby transforming into pathogenic drivers. This observation underscores that, in complex environments characterized by multiple concurrent perturbations (such as antibiotic exposure, mucosal barrier injury, and alloimmunity), FMT as a community-level intervention may require a more precisely engineered approach.

#### 4.4.2 The ARES trial and MaaT013

In contrast, MaaT013, a pooled, high-richness microbiota biotherapeutic, has demonstrated more promising results in a rigorously designed clinical trial. The single-arm, open-label Phase III ARES trial evaluated MaaT013 as third-line therapy in 66 patients with severe GI-aGVHD refractory to both corticosteroids and ruxolitinib (98). Among enrolled patients who received three consecutive doses of MaaT013, the gastrointestinal response rate reached 62%, and the 1-year overall survival rate was 54%. These outcomes are clinically meaningful in this highly refractory patient population and suggest that standardized, high-richness microbiota restoration may reverse disease progression even in cases of multidrug-resistant severe aGVHD.

#### 4.4.3 Tissue-targeted cytokine therapy

F-652, a recombinant human IL-22 fusion protein, represents a novel tissue-supportive strategy that targets epithelial repair rather than broad immunosuppression. In a multicenter Phase II trial of 27 patients with newly diagnosed lower GI-aGVHD, F-652 in combination with corticosteroids achieved a Day 28 response rate of 70% (99). Notably, F-652 has also shown preliminary efficacy in the steroid-refractory setting, with case reports documenting partial responses in patients who had failed multiple prior lines of therapy including ruxolitinib, accompanied by resolution of GI bleeding and bacteremia (100). The mechanism involves promotion of intestinal epithelial regeneration, enhancement of antimicrobial peptide secretion, and modulation of gut microbiota composition, including expansion of protective Blautia species.

#### 4.4.4 Novel immunomodulatory agents

Xenopax, a humanized IL-2 receptor antagonist, has been approved for the treatment of SR-aGVHD (101). The multicenter retrospective RELAX study demonstrated that Xenopax is both effective and well tolerated in real-world clinical settings. Additionally, preliminary data from EBMT 2025 suggest that Xenopax effectively prevents severe GVHD in haploidentical HSCT for severe aplastic anemia without compromising engraftment or increasing severe infection risk (102).

Itolizumab is a humanized anti-CD6 monoclonal antibody that selectively targets the CD6-ALCAM pathway to downregulate pathogenic T effector cells while sparing regulatory T cells (103). In the phase 1b/2 EQUATE trial, 30 patients with grade III-IV aGVHD received itolizumab plus corticosteroids, achieving a day-29 OR rate of 63% and CR rate of 43%, with rapid responses by day 15 and sustained steroid tapering (70% reduction by day 29) (104). The future clinical development of itolizumab in aGVHD remains uncertain pending further regulatory discussions and potential partnership opportunities.

α1-Antitrypsin is a serine protease inhibitor with pleiotropic anti-inflammatory, anti-apoptotic, and immunomodulatory properties. Preclinical studies demonstrated that AAT suppresses IL-32 activation and reduces pro-inflammatory cytokines (TNF-α, IL-1β), thereby attenuating GVHD severity while preserving the GVL effect. Clinically, Marcondes (105) reported that AAT salvage therapy achieved an overall response rate of 66.5% with 33.3% complete response in 12 patients with steroid-refractory aGVHD. In a subsequent phase II trial, Magenau (106) treated 40 patients with steroid-refractory aGVHD with AAT (60 mg/kg twice weekly for up to 4 weeks), achieving a day-28 overall response rate of 65% with minimal toxicity. However, a biomarker-guided preemptive study by Gergoudis (107) in 30 high-risk patients (elevated REG3α and ST2) did not demonstrate a significant reduction in steroid-refractory GVHD incidence compared with contemporaneous controls (20% vs. 14%, P = 0.56).

#### 4.4.5 Preclinical therapeutic targets with translational potential

Several promising targets identified through mechanistic studies remain in the preclinical stage and await formal clinical translation. Recombinant LCN2 protein has demonstrated efficacy in murine models by ameliorating aGVHD without compromising GVL effects, operating through the LCN2-SLC22A17-IGF-1R axis to promote IL-10 production by macrophages (64). R-spondin1, a Wnt agonist, promotes regeneration of intestinal stem cells and Paneth cells, attenuating GVHD severity while restoring microbial diversity in preclinical models (108, 109). Teduglutide, a GLP-2 analog, promotes expansion of intestinal stem cells and has shown preliminary evidence of improving microbial diversity and alleviating intestinal injury (110). These agents represent a pipeline of mechanism-driven therapeutics that may enter clinical trials in the coming years.

Mesenchymal stem cells (MSCs) exhibit broad immunomodulatory capacity through regulation of T and B lymphocytes, natural killer cells, and dendritic cells, alongside promotion of regulatory T-cell expansion and tissue repair. Remestemcel-L, an ex vivo culture-expanded allogeneic MSC product, achieved a day-28 OR rate of 70.4% in a phase III trial of 54 pediatric patients with primary SR-aGVHD, significantly exceeding the 45% historical control rate (*P* = 0.0003), with durable responses maintained through day 100 (111). A large Chinese phase III trial further showed that MSCs combined with basiliximab and calcineurin inhibitors improved day-28 and day-56 durable responses, and reduced 2-year cGVHD incidence (39.5% vs. 62.7%) compared with controls (112). These findings establish MSC-based therapy as a validated strategy for steroid-refractory aGVHD, with ongoing efforts to optimize cell source, dosing, and timing.

### 4.5 A risk-stratified diagnostic and therapeutic algorithm

**Figure 2:**
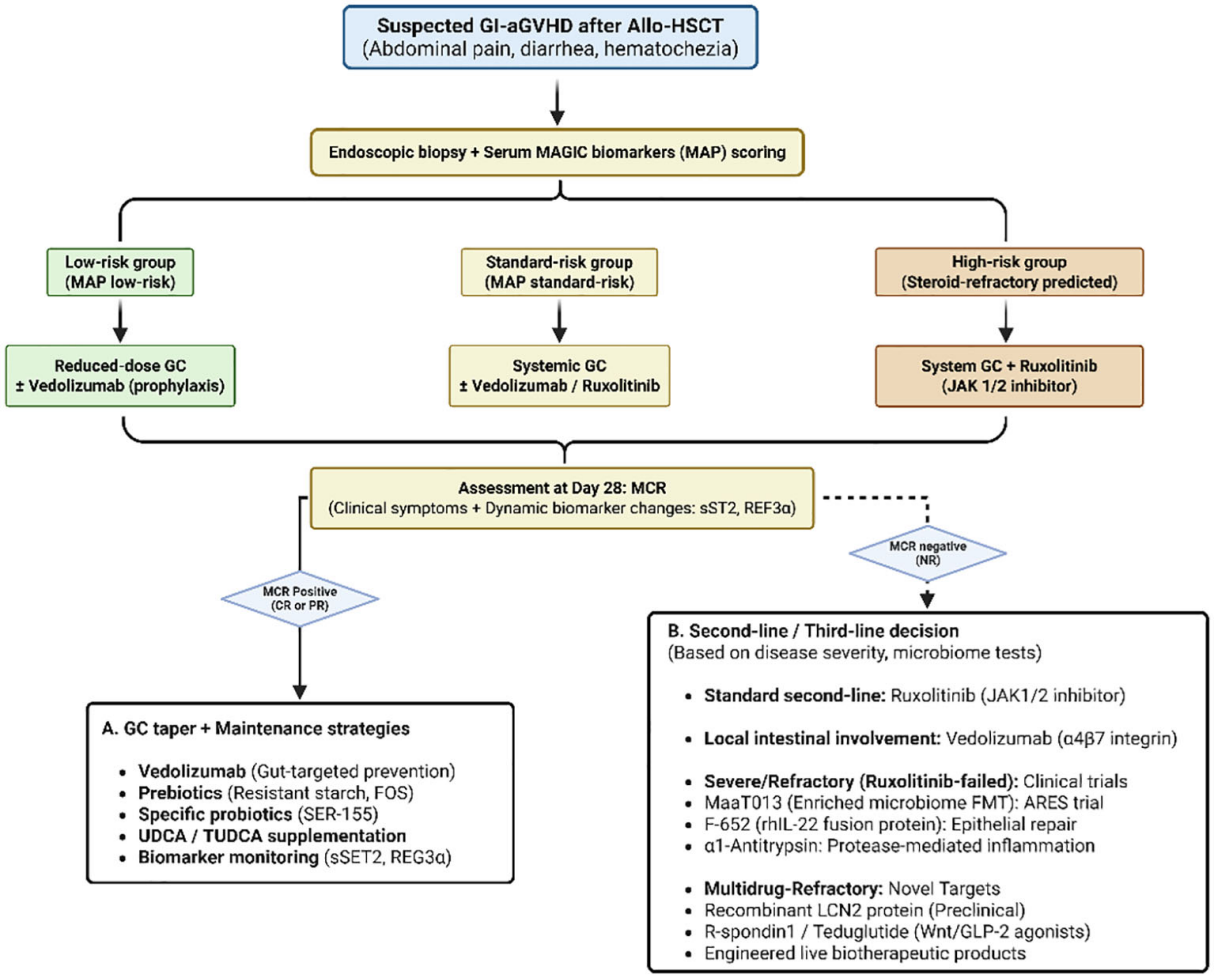
Risk-stratified diagnostic and therapeutic algorithm for suspected GI-GVHD after allo-HSCT. The algorithm comprises three sequential decision nodes (1): Pre-transplant risk stratification, based on microbiota diversity and MAGIC biomarker baseline (2); First-line therapy selection, wherein GC are administered, with vedolizumab or ruxolitinib added according to the MAP risk category (3); Day 28 response assessment, utilizing the MCR, which integrates clinical response (GVHD grading per MAGIC criteria) with biomarker dynamics (sST2 and REG3α). Patients achieving MCR-defined CR or PR at day 28 may proceed to GC tapering under continued biomarker surveillance. Abbreviations: GI-aGVHD, gastrointestinal acute graft-versus-host disease; Allo-HSCT, allogeneic hematopoietic stem cell transplantation; MAP, MAGIC algorithm probability; GC, glucocorticoid; MCR, MAGIC composite response; FOS, fructo-oligosaccharides; JAK, Janus kinase; FMT, fecal microbiota transplantation; LCN2, lipocalin-2; TUDCA, tauroursodeoxycholic acid.

The emergence of these diverse therapeutic modalities, alongside targeted agents such as ruxolitinib and vedolizumab, is facilitating the construction of a multifaceted and individualized framework for the management of aGVHD. A risk-stratified diagnostic and therapeutic algorithm for suspected GI-aGVHD is provided in **Figure 2**, integrating MAGIC biomarker assessment, clinical staging, and mechanism-based therapeutic selection to guide precision management across the continuum from prophylaxis to refractory disease. Critical to this algorithm is the day 28 assessment node, operationalized via the MCR, which integrates clinical GVHD grading with longitudinal biomarker trajectories (sST2 and REG3α) (17). Patients achieving MCR-defined response (complete or partial) should proceed to glucocorticoid tapering, with continued biomarker surveillance to detect early relapse. Conversely, MCR-defined non-responders require immediate therapeutic escalation. In ruxolitinib-naïve patients, JAK1/2 inhibition constitutes the established second-line standard of care (113). For patients with ruxolitinib-refractory or intolerant disease, mechanism-matched alternatives are prioritized, including MaaT013 (98) for microbiota restoration, F-652 (100) for epithelial repair, and α1-antitrypsin (106) for protease-mediated inflammation. For multidrug-refractory cases, enrollment in clinical trials evaluating engineered live biotherapeutic products, recombinant LCN2 (64)immunomodulation, or Wnt/GLP-2 pathway agonist (108, 114) is strongly encouraged. This sequential, biomarker-anchored strategy ensures that therapeutic intensity escalates commensurately with refractoriness, while avoiding premature exposure to agents with overlapping mechanisms.

## 5 Future perspectives and translational directions

### 5.1 Engineering strategies for precision microbiota intervention

Building on the *Enterococcus*–MHC-II axis, the *Bacteroides* T6SS–bile acid circuit, and butyrate-mediated Treg homeostasis, precision microbiota-targeted interventions should advance along three tiers. First, the development of function-oriented, engineered microbial consortia or single-strain therapeutics (116). Through synthetic biology approaches, commensal bacteria can be directionally modified (such as augmenting T6SS activity in Bacteroides ovatus to antagonize mucus-degrading species (39)). Alternatively, genes encoding butyrate synthesis enzymes can be introduced into safe chassis strains, thereby enabling the controlled, local release of key protective metabolites within the intestinal epithelium and circumventing the potential colonization risks posed by live microbes in immunocompromised hosts (98). Second, the construction of a multidimensional patient stratification system integrating microbial profiles, metabolomic signatures, and immune landscapes to guide the precise timing of individualized interventions. Within prospective transplant cohorts, the dynamic trajectories of MAGIC biomarkers (sST2, REG3α) (73), fecal microbial diversity indices (37), and targeted metabolomic data on secondary bile acids and SCFAs should be amalgamated to establish machine learning models capable of predicting the likelihood of benefit from specific microbiome-based therapies. Third, leveraging the aforementioned stratification framework to execute multicenter, randomized controlled trials that rigorously evaluate the preventive and therapeutic efficacy of standardized, high-richness live biotherapeutic products and key postbiotics (such as sodium butyrate, tauroursodeoxycholic acid) within well-defined patient subgroups (58).

### 5.2 Clinical translation of novel therapeutic targets

Among the preclinical targets summarized in Section 4.4, several have advanced toward clinical evaluation. The LCN2-SLC22A17-IGF-1R axis, initially characterized in murine GI-aGVHD models (64), has informed the rationale for exploring neutrophil-derived immunomodulatory pathways in early-phase trials. Concurrently, the Wnt agonist R-spondin1 and the GLP-2 analog teduglutide, both demonstrating intestinal stem cell protective effects in preclinical settings (109, 115), have established proof-of-concept for epithelial-directed strategies, though formal clinical trials in aGVHD remain pending. The translational trajectory of these agents will depend on biomarker-guided patient selection and the development of validated manufacturing protocols for biologic therapeutics.

### 5.3 Biomarker-driven redesign of clinical trials

It is important to emphasize that, in everyday clinical practice, the diagnosis and grading of aGVHD continue to rely primarily on clinical assessment in the majority of transplant centers, with endoscopic biopsy and histopathological confirmation serving as supplementary rather than routine tools (2). Against this backdrop, the introduction of the MAGIC composite response metric signifies a new era in endpoint design for aGVHD clinical trials (16, 17). Integrating biomarkers into the efficacy assessment framework not only permits more accurate prognostication of long-term survival outcomes but also enables the identification of low-risk patients who may genuinely benefit from reduced-intensity immunosuppressive strategies, thereby averting unnecessary overtreatment (73, 75, 77). Future clinical investigations should prospectively incorporate MAGIC biomarkers as both stratification factors and endpoint evaluation tools to expedite the validation and adoption of effective therapies.

### 5.4 Special considerations in non-malignant disorders such as aplastic anemia

For patients with non-malignant disorders such as AA undergoing allo-HSCT, the therapeutic objective, given the absence of a requisite GVT effect, should center on minimizing GVHD risk, reducing treatment-related complications, and optimizing long-term quality of life (117). The 2024 ASTCT (118) evidence-based guidelines for allo-HSCT in SAA recommend calcineurin inhibitor (CNI) plus methotrexate or post-transplant cyclophosphamide (PTCy)-based GVHD prophylaxis for matched related or matched unrelated donor transplants, with bone marrow as the preferred stem-cell source and rabbit ATG as the preferred serotherapy.

Emerging evidence supports the integration of novel agents into GVHD prophylaxis regimens for non-malignant diseases. A retrospective study demonstrated that the addition of ruxolitinib to standard GVHD prophylaxis significantly reduced grade II-IV aGVHD incidence in SAA patients undergoing allo-HSCT, accelerated platelet engraftment, enhanced Treg recovery, and improved 1-year GRFS (117). Additionally, preliminary data from EBMT 2025 suggest that the humanized anti-CD25 monoclonal antibody Xenopax effectively prevents severe GVHD in haploidentical HSCT for SAA without compromising engraftment or increasing severe infection risk (102). The AbaCyS protocol (abatacept, PTCy, and sirolimus) has also shown promising results in SAA and thalassemia, with extremely low incidences of aGVHD and cGVHD (119). Given the absence of GVT requirements and the favorable long-term survival trajectory characteristic of non-malignant disorders, a critical unmet need exists for the formulation of dedicated, evidence-based GVHD guidelines tailored to this patient population. Such guidelines must account for disease-specific considerations, including negligible relapse risk, heightened emphasis on long-term quality of life, and distinct patterns of immune reconstitution, and prospectively evaluate whether reduced-intensity immunosuppressive regimens or microbiota-directed interventions may be safely de-escalated in this context.

## 6 Conclusion

In conclusion, this review provides a translational synthesis that integrates the microbiota-immune-epithelial pathogenic axis, MAGIC biomarker-driven risk stratification, and targeted prevention and treatment for GI-GVHD after allo-HSCT, positioning microbiota-directed strategies as core interventions with particular attention to nonmalignant disorders such as AA. Recognizing that the self-amplifying cycle of immune injury, barrier disruption, and dysbiosis precludes a single-agent solution, we articulate three strategic priorities: combinatorial or sequential therapies that concurrently target immune attack, fortify epithelial integrity, and restore microbial eubiosis; prospective application of MAGIC biomarkers to guide real-time therapeutic decisions, including immunosuppression de-escalation in low-risk patients and preemptive microbiome-directed intervention in high-risk subsets; and a transition from empirical fecal microbiota transplantation to precision microbiome engineering using defined consortia and postbiotics such as butyrate and secondary bile acids. By offering a risk-stratified diagnostic and therapeutic algorithm and providing a prioritized catalog of actionable targets, this work charts a clear agenda to disrupt the pathogenic cycle of GI-GVHD through synergistic modulation of immunity, the microbiome, and the epithelial barrier, with the ultimate goal of reducing non-relapse mortality and improving long-term outcomes for transplant recipients.

## Glossary

AA: Aplastic Anemia
aGVHD: Acute Graft-versus-Host Disease
AhR: Aryl Hydrocarbon Receptor
allo-HSCT: Allogeneic Hematopoietic Stem Cell Transplantation
AML: Acute Myeloid Leukemia
APC: Antigen-Presenting Cell
APOE: Apolipoprotein E
AREG: Amphiregulin
Bas: Bile Acids
cDC: conventional Dendritic Cell
CEACAM1: Carcinoembryonic Antigen-Related Cell Adhesion Molecule 1
DAMPs: Damage-Associated Molecular Patterns
FMT: Fecal Microbiota Transplantation
FOS: Fructo-Oligosaccharides
FOXP3: Forkhead Box P3
FXR: Farnesoid X Receptor
GC: Glucocorticoid
GFO: Galacto-Fructo-Oligosaccharides
GI: Gastrointestinal
GI-aGVHD: Gastrointestinal Acute Graft-versus-Host Disease
GVHD: Graft-versus-Host Disease
GVL: Graft-versus-Leukemia
GVT: Graft-versus-Tumor
HDAC: Histone Deacetylase
IEC: Intestinal Epithelial Cell
IFN-γ: Interferon-gamma
IL: Interleukin
ILC2s: Type 2 Innate Lymphoid Cells
ISC: Intestinal Stem Cell
LCA: Lithocholic Acid
LCN2: Lipocalin-2
LPS: Lipopolysaccharide
MAGIC: Mount Sinai Acute GVHD International Consortium
MAP: MAGIC Algorithm Probability
MCR: MAGIC Composite Response
MCS: MAGIC Composite Score
MHC-II: Major Histocompatibility Complex Class II
NLRP3: NLR Family Pyrin Domain Containing 3
NRM: Non-Relapse Mortality
OS: Overall Survival
PAMPs: Pathogen-Associated Molecular Patterns
pTreg: peripheral regulatory T cell
RCT: Randomized Controlled Trial
REG3α: Regenerating Islet-Derived 3-alpha’
SAA: Severe Aplastic Anemia
SCFAs: Short-Chain Fatty Acids
SOCS1: Suppressor of Cytokine Signaling 1
SR-aGVHD: Steroid-Refractory Acute Graft-versus-Host Disease
sST2: soluble Suppression of Tumorigenicity 2
T6SS: Type VI Secretion System
TGR5: G Protein-Coupled Bile Acid Receptor 1
TMAO: Trimethylamine N-Oxide
TUDCA: Tauroursodeoxycholic Acid
UDCA: Ursodeoxycholic Acid
